# Genetic Circuits for Feedback Control of Gamma-Aminobutyric Acid Biosynthesis in Probiotic *Escherichia coli* Nissle 1917

**DOI:** 10.3390/metabo14010044

**Published:** 2024-01-11

**Authors:** Matthew Lebovich, Marcos A. Lora, Jared Gracia-David, Lauren B. Andrews

**Affiliations:** 1 Department of Chemical Engineering, University of Massachusetts Amherst, Amherst, MA 01003, USA; 2 Biotechnology Training Program, University of Massachusetts Amherst, Amherst, MA 01003, USA; 3 Department of Biology, Amherst College, Amherst, MA 01002, USA; 4 Molecular and Cellular Biology Graduate Program, University of Massachusetts Amherst, Amherst, MA 01003, USA

**Keywords:** genetically encoded biosensor, synthetic gene regulation, living therapeutic, gut microbial metabolites, depression, neurotransmitter, gamma-aminobutyric acid (GABA)

## Abstract

Engineered microorganisms such as the probiotic strain *Escherichia coli* Nissle 1917 (EcN) offer a strategy to sense and modulate the concentration of metabolites or therapeutics in the gastrointestinal tract. Here, we present an approach to regulate the production of the depression-associated metabolite gamma-aminobutyric acid (GABA) in EcN using genetic circuits that implement negative feedback. We engineered EcN to produce GABA by overexpressing glutamate decarboxylase and applied an intracellular GABA biosensor to identify growth conditions that improve GABA biosynthesis. We next employed characterized genetically encoded NOT gates to construct genetic circuits with layered feedback to control the rate of GABA biosynthesis and the concentration of GABA produced. Looking ahead, this approach may be utilized to design feedback control of microbial metabolite biosynthesis to achieve designable smart microbes that act as living therapeutics.

## 1. Introduction

Gamma-aminobutyric acid (GABA) is a non-essential amino acid produced by bacteria found in the human gastrointestinal tract [1]. It is a neurotransmitter that has been shown to affect neurological conditions including mood and sleep disorders [2,3] as well as anxiety, depression, and epilepsy [2,4,5]. This has led to interest in being able to regulate the biosynthesis and availability of GABA in vivo, including in the human gut, where it can exert neuromodulatory effects via the gut–brain axis [6,7,8]. Engineered probiotic bacteria, such as *Escherichia coli* Nissle 1917 (EcN), have been proposed as a next-generation strategy to produce, deliver, and modulate microbially derived metabolites in the gut. Wildtype (nonengineered) EcN has been used clinically as a probiotic and therapy for ulcerative colitis (trade name of Mutaflor^®^) [9,10] and has been generally regarded as safe. Engineered EcN has been developed as a bacterial therapeutic strategy to modulate metabolite concentrations in the gut, such as a strain developed to metabolize phenylalanine for patients with the inherited metabolic disorder phenylketonuria [11,12], or to produce a variety of biomolecules [13,14,15,16]. Controlling the concentration of a metabolite, such as GABA, in vivo would require synthetic regulation. Various strategies have been modeled and employed to introduce feedback and genetic circuits for cells to autonomously self-regulate the output level of a product in different cell types [17,18,19,20]. Here, we sought to develop a model-guided approach to design feedback circuits to control metabolite biosynthesis in EcN using characterized genetic circuit components. For this work, we chose GABA biosynthesis as our test case and used a set of modular repressor-based transcriptional logic (i.e., NOT) gates to construct feedback circuits [21,22,23]. These transcriptional NOT gates were characterized in EcN by our group and were demonstrated as an approach to building complex genetic circuits using design algorithms in EcN [23]. Here, we sought to test whether we could regulate GABA biosynthesis in EcN through feedback control implemented via genetic circuits designed and built from these logic gates.

To regulate the concentration of GABA, EcN cells also need to have machinery to sense and produce the GABA metabolite. In other work, we previously developed and reported the first known GABA biosensor for EcN that uses an allosteric transcription factor regulated by GABA (GabR) from *Bacillus subtilis* 168 [24] and a synthetic P_Gab_ promoter (P_Gab105_) engineered for EcN [25], which is the transcriptional output signal of the biosensor. We optimized the sensor design for 138-fold activation in EcN and showed that it specifically senses GABA with negligible activation to structurally similar metabolites [25]. Therefore, we chose to use this GABA biosensor in this work. For the biosynthesis of GABA, other studies have engineered improved GABA production in *E. coli* by overexpressing glutamate decarboxylases GadA and GadB, which convert L-glutamate into GABA, and by overexpressing the glutamate/GABA antiporter GadC [26,27]. Biosensors have been widely used for high-throughput screening to guide pathway optimization and metabolic engineering [28,29,30]. Therefore, we sought to apply this approach to engineer GABA biosynthesis and feedback control in probiotic EcN.

**Figure 1:**
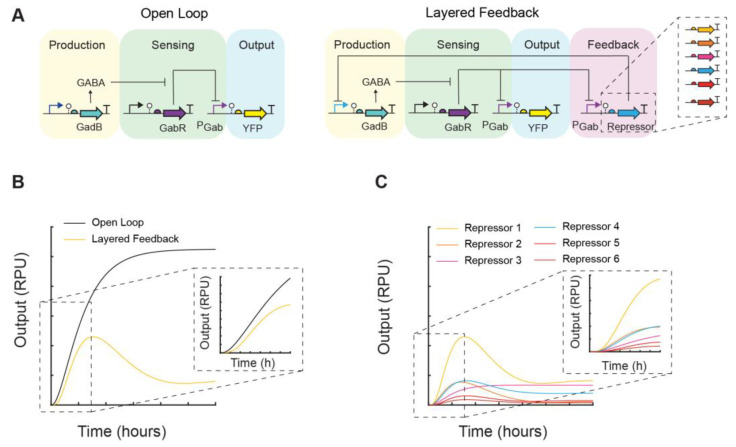
Theoretical framework for self-regulation of GABA biosynthesis in EcN with designable feedback. (**A**) The proposed open-loop and layered-feedback regulatory networks for GABA production are shown. In the open-loop production module, the expression of the enzyme GadB is induced and subsequently produces GABA. In the sensing module, GABA induces the P_Gab_ promoter (purple) of the GABA biosensor regulated by GabR (purple). The P_Gab_ promoter, in turn, is the transcriptional driver for the output module, such as a yellow fluorescent protein (YFP) reporter. In the layered-feedback design, the sensor output drives the expression of a repressor-based NOT gate that controls the expression of the GadB enzyme, creating a negative feedback loop. These transcriptional NOT gates are interchangeable, and those with different input–output responses can control the dynamics of GABA production. Mathematical models were constructed to simulate the output (P_Gab_) of these regulatory networks (Methods). (**B**) Simulation results comparing the output of the open loop and a layered-feedback design. (**C**) Simulation results comparing layered-feedback designs containing six different repressor NOT gates. The output is reported using standard relative promoter units (RPU) (Methods).

Here, we use a biosensor-assisted approach for the metabolic engineering of GABA biosynthesis in EcN and integrate genetic circuits to regulate GABA production via feedback control. First, we perform genome editing to engineer EcN for GABA biosynthesis. Then, using a GABA biosensor, we screen the GABA production of these engineered EcN strains and also investigate the effects of pH and culturing conditions on GABA biosynthesis. Lastly, we construct and assay open-loop and feedback genetic circuits for the regulation of GABA production by EcN. Using modular, genetically encoded NOT gates to construct feedback circuits, we were able to monitor and regulate the rate of GABA production (Figure 1).

## 2. Materials and Methods

### 2.1. Strains, Media, and Inducers

*E. coli* was used for experimentally assaying all genetic constructs. *E. coli* NEB 5-alpha (New England Biolabs, Ipswich, MA, USA) and LB Miller medium (Fisher Scientific, Waltham, MA, USA) was used for cloning. Cultures for assays, including overnight cultures, were grown in M9 media (Sigma-Aldrich, Burlington, MA, USA; 6.78 g/L Na_2_HPO_4_, 3 g/L KH_2_PO_4_, 1 g/L NH_4_Cl, 0.5 g/L NaCl final concentration) with 0.34 g/L thiamine hydrochloride (Sigma-Aldrich), 0.2% *w*/*v* casamino acids (Acros), 2 mM MgSO_4_ (Sigma-Aldrich), 0.1 mM CaCl_2_ (Sigma-Aldrich), and 0.4% *w*/*v* D-glucose (Sigma-Aldrich). Strains were assayed in M9 media with 35 g/L monosodium glutamate (MSG; Sigma-Aldrich). The pH was adjusted using hydrochloric acid (Fisher). The antibiotics used for selection were 50 μg/mL kanamycin (GoldBio, St. Louis, MO, USA), 100 μg/mL ampicillin (GoldBio), and 5 μg/mL chloramphenicol (GoldBio). The inducers used were anhydrotetracycline hydrochloride (aTc; Sigma-Aldrich) and gamma-aminobutyric acid (GABA; Sigma-Aldrich). GABA was stored as an aqueous solution, and aTc was dissolved in 100% ethanol.

### 2.2. Construction of GABA Production Plasmids

The construction of the *gadB* containing plasmids was performed in two steps. First, part plasmids containing either a promoter or a multi-part construct (ribozyme, RBS, gene coding sequence, terminator) were combined into a transcription unit plasmid in a Type IIS DNA assembly reaction using BsaI-HFv2 (New England Biolabs). The destination vector (p15A origin of replication, ampicillin resistance) was supplied as a purified PCR product, and the genetic parts used were purified part plasmids. In the second Type IIS DNA assembly reaction using BbsI (New England Biolabs), these transcriptional unit constructs were assembled into the backbone plasmid pML3001 [25], which contains kanamycin resistance, the p15A low-copy origin of replication, and regulators for the sensors (*gabR* and *tetR*). The multi-part construct plasmids containing a ribozyme, synthetic RBS, *gadB*, and terminator were constructed through being inserted into a plasmid backbone containing kanamycin resistance and the colE1 ORI via a Type IIS DNA assembly reaction with the BbsI enzyme. The *gadB* gene was amplified from *E. coli* MG1655 genomic DNA. All parts and plasmids used in this work can be found in Tables S1 and S2.

Type IIS DNA assembly reactions were performed in 5 μL total volume containing 20 fmol of each purified part or transcription unit plasmid, 10 fmol of the purified destination vector PCR product, 5 U of the appropriate Type IIS restriction enzyme, and 125 U T4 DNA ligase (2000 U/µL; New England Biolabs) in 1X T4 DNA Ligase Buffer (New England Biolabs). The reaction mixture was incubated in a thermal cycler (Bio-Rad, Hercules, CA, USA, C1000 thermal cycler, 105 °C lid) with the protocol: 37 °C for 6 h, followed by 50 °C for 30 min, and inactivation at 80 °C for 15 min. Then, 2 μL of the assembly reaction was transformed into 5 μL chemically competent cells (*E. coli* NEB 5-alpha, New England Biolabs). Circuit constructs were analyzed using PCR. All transcriptional unit plasmids were sequenced via Sanger sequencing (Azenta formerly Genewiz, Burlington, MA, USA). Plasmids used in this work were deposited to Addgene.

### 2.3. Construction of the gabTP Deletion in EcN

The genes *gabT* and *gabP* were deleted from the EcN genome using the pSIJ8 plasmid [31] containing the lambda Red recombineering system and ampicillin resistance (Addgene plasmid #68122). The plasmid was transformed into EcN via electroporation. Using the pKD3 plasmid [32] as the template, 500 bp homology arms (first 500 bp of *gabT* and last 500 bp of *gabP*) were added to either side of the chloramphenicol cassette to create pKD3-gab. Following the protocol previously described [31], EcN cells harboring pSIJ8 were grown to an OD_600_ of approximately 0.3 in LB media (Fisher). The lambda Red proteins were induced by adding L-arabinose to a final concentration of 15 mM and growing the cells for an additional 45 min. The cells were then made electrocompetent, and a purified PCR product of pKD3-gab containing the homology arms and chloramphenicol cassette was transformed into EcN harboring pSIJ8 via electroporation. The cells were recovered in SOC for 2 h at 30 °C and plated on an LB agar plate containing chloramphenicol and ampicillin. The plate was incubated at 30 °C overnight. A single colony was streak-purified at 30 °C. To remove pSIJ8, the new strain was grown in liquid culture at 37 °C, and a sample was streaked onto an agar plate with plate chloramphenicol. This last step was repeated until a colony from the chloramphenicol plate showed no growth on an ampicillin plate.

### 2.4. GABA Production Assays

An overnight culture for each strain was started in M9 media (pH 7.0) from a freezer stock stored at −80 °C. Cells were inoculated at an OD_600_ of 5 × 10^−5^ from an overnight culture and grown in M9 media with the appropriate antibiotics in 14 mL culture tubes (Fisher). Samples were grown in M9 media (pH 5.5 unless otherwise indicated) with 35 g/L MSG with appropriate antibiotics and inducers. Flask cultures were grown in 20 mL of M9 media in 125 mL Erlenmeyer flasks. Tube cultures were grown in 7 mL of M9 media in a 14 mL culture tube (Fisher). Plate cultures were grown in 200 μL of M9 media in the well of a 96-well U-bottom microtiter plate (Corning Costar, Corning, NY, USA). Multiple wells from the same overnight were inoculated in plates to provide enough sample to analyze. All samples were grown at 37 °C. Flasks and tubes were shaken at 250 rpm and 37 °C in a shaking incubator (Innova 44R, Eppendorf, Enfield, CT, USA). Plates were incubated in an ELMI DTS-4 digital thermostatic microplate shaker at 1000 rpm. After five hours of growth, a sample of each culture was taken every hour and incubated for 30 min at room temperature in PBS with 2 mg/mL kanamycin before fluorescence was measured using flow cytometry.

### 2.5. Flow Cytometry Analysis

Cell fluorescence was measured using a BD Accuri C6 flow cytometer using a 480 nm blue laser and the FL1-A detection channel. The data for each sample were collected with a flow rate of less than 1000 events/s and at least 10,000 gated events collected per sample. For data analysis, the events were gated with a gate for cell-sized particles using FlowJo software (version 10). The median cell fluorescence for each sample was calculated using FlowJo. Histograms of cell fluorescence were generated using FlowJo. In all flow cytometry assays, the autofluorescence of wildtype EcN cells and the fluorescence of EcN cells containing the RPU standard plasmid were measured for samples from three separate colonies using identical dilutions and growth conditions as the assayed constructs.

The measured cell fluorescence in arbitrary units was converted to relative promoter units (RPU) as previously described [21] and using equation 1 with the arbitrary unit values for the sample cell fluorescence (*YFP*), autofluorescence of wildtype EcN cells (*YFP_o_*), and fluorescence of EcN cells containing the RPU standard plasmid pAN1717 [21] (*YFP_RPU_*), which constitutively expresses eYFP. The sample fluorescence on each day’s experiment was converted to RPU. For each experiment, the average of the cell fluorescence from three separate colonies was used for the autofluorescence and RPU plasmid standard. The limit of detection was set to 0.001 RPU, and an output below this cutoff was set to this minimum value.

RPU = (*YFP* − *YFP_o_*)/(*YFP_RPU_* − *YFP_o_*) (1)

### 2.6. Modeling

The following coupled differential equations were used to model the mRNA concentration of the transcript for GadB expression (*M_B_*), concentration of GadB enzyme (*B*), concentration of GABA (*G*), transcriptional output of the P_Gab_ promoter (*μ_G_*), and transcriptional output of the promoter transcribing *gadB* (*Q_r_*) and are based on a previously published model [33]:

(2) $$\frac{dM_{B}}{dt}=Q_{r}\xi -\gamma M_{B}$$

(3) $$\frac{dB}{dt}=\alpha_{B}M_{B}-\beta_{B}B$$

(4) $$\frac{dG}{dt}=kSB-\beta_{G}G$$

(5) $$\frac{d\mu_{G}}{dt}=\alpha_{\mu_{G}}\left[y_{min\mu_{G}}+(y_{max\mu_{G}}-y_{min\mu_{G}})\frac{{K_{\mu_{G}}}^{n_{\mu_{G}}}}{{K_{\mu_{G}}}^{n_{\mu_{G}}}{+G}^{n_{\mu G}}}\right]-\beta_{\mu_{G}}\mu_{G}$$

(6) $$\frac{dQ_{r}}{dt}=\gamma (\left[y_{min_{r}}+(y_{max_{r}}-y_{min_{r}})\frac{{K_{r}}^{n_{r}}}{{K_{r}}^{n_{r}}{+\mu_{G}}^{n_{r}}}\right]-Q_{r})$$

In the layered-feedback circuits, *Q_r_* is the output of the repressor NOT gate, as shown in Equation (6). In the case of the open-loop regulatory network, *Q_r_* was set to the maximum output of P_Tet_, and equation 6 was set to zero. The value of *μ_G_* is the output of P_Gab_ in the GABA sensor. The parameters *α* and *β* represent the production and degradation rate constants of their respective species. The degradation rate of mRNA, *γ*, has been set to 0.025 min^−1^ as in previous work [33]. A conversion factor *ξ* has been set to 0.025 [mRNA] min^−1^ RPU^−1^ with *Q_r_* = *M_Qr_ξ^−1^γ* [33]. The parameters *y_min_*, *y_max_*, *K*, and *n* are the values for the corresponding GABA sensor or NOT gate’s response function [23]. Equations were solved using the ode45 function in MATLAB. Lists of all parameters used are given in Tables S3 and S4.

## 3. Results

### 3.1. Effects of Growth Conditions on GABA Production

**Figure 2:**
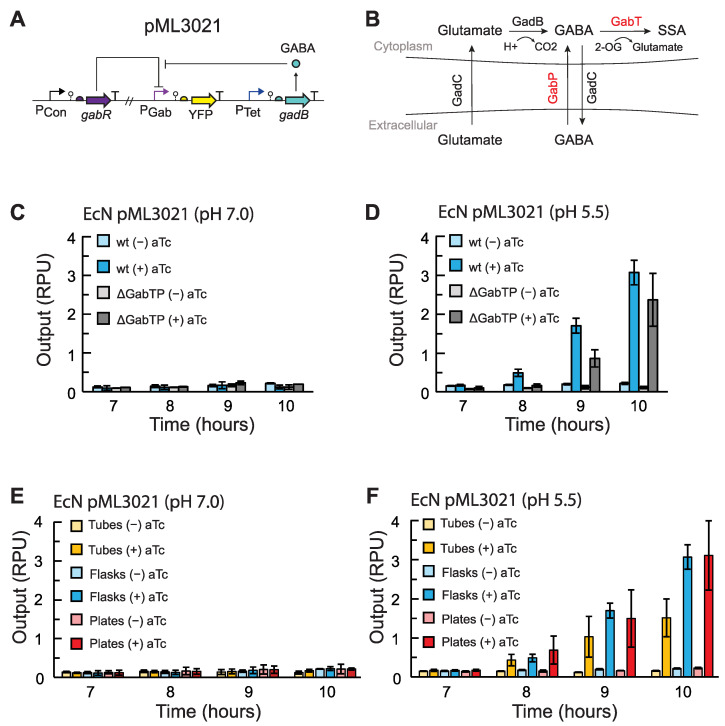
Use of a GABA biosensor to screen strains and conditions for GABA production in EcN. (**A**) The GABA production plasmid pML3021 has inducible GadB expression. GadB produces GABA, which induces P_Gab_ and the expression of eYFP via the GABA sensor regulated by GabR. (**B**) L-glutamate is transported into the cell via the antiporter GadC and then converted into GABA by GadB. GABA can be degraded into succinate semialdehyde (SSA) via GabT (red). 2-oxogluterate (2-OG) is consumed in the reaction. GABA can also be imported into the cell by GabP (red). (**C**) GABA production assays for EcN without chromosomal editing (wt) and Δ*gabTP* chromosomal deletion strains harboring pML3021. Cells were grown in flask cultures without aTc (−) or with aTc added to a final concentration 2 ng/mL (+) at an initial pH = 7.0 or (**D**) initial pH 5.5. (**E**) GABA production assays for EcN wt harboring pML3021 grown in flasks, tubes, or plates without aTc (−) or with 2 ng/mL aTc (+) at an initial pH = 7.0 or (**F**) initial pH 5.5. All cultures were grown in M9 media supplemented with 35 g/L MSG and inoculated at an OD_600_ of 5 × 10^−5^. After 7 h of growth, cell fluorescence was measured via flow cytometry at each time point. Fluorescence was converted into relative promoter units (RPU) (Methods). Bars represent the average of the measured median of a population of at least 10,000 cells assayed in three identical experiments performed on three separate days. Error bars represent the standard deviation.

We first aimed to construct an EcN strain for GABA production and examine how changes in gene expression affected GABA production. To detect GABA, the previously developed GABA biosensor for EcN was utilized for screening [25]. A yellow fluorescent protein (eYFP) reporter was placed under the control of the P_Gab_ sensor output promoter for all constructs. In addition to containing the GABA sensor, the plasmid pML3021 expressed *E. coli* glutamate decarboxylase (GadB) [26] under the control of the inducible promoter P_Tet_ (Figure 2A). In this system, GadB converts L-glutamate into GABA [26], which then induces the P_Gab_ promoter. The plasmid pML3021 was transformed into wildtype EcN, as well as a strain with the chromosomal *gabT* and *gabP* genes deleted (EcN Δ*gabTP*). The deletion of *gabT* and *gabP* has been shown in previous work to increase GABA production [27]. GabT digests GABA into succinate semialdehyde, and GabP imports GABA [27], which may be beneficial to delete if the end goal is to export GABA in vivo (Figure 2B). Both strains were grown in 20 mL flask cultures containing M9 media supplemented with 35 g/L monosodium glutamate (MSG). To induce expression of GadB, 2 ng/mL anhydrotetracycline (aTc) was added to the appropriate samples. Samples were grown at 37 °C for 7 h, after which an aliquot was taken every hour. The single-cell fluorescence was measured via flow cytometry and converted into RPU (Methods).

When GadB was induced in media at pH 7.0, no significant change in P_Gab_ output was observed for any strains, which indicates negligible GABA production at all time points (Figure 2C). The output observed was equivalent to the basal activity of the P_Gab_ promoter, as previously reported for the sensor [25]. This result is in agreement with prior studies reporting that GadB, which is notably part of an *E. coli* acid resistance system, is inactive at neutral pH [34,35]. When we lowered the initial pH of the medium to 5.5, we observed P_Gab_ activation beginning at 8 h after induction with aTc, indicating GABA production (Figure 2D). In our experiments, deleting *gabT* and *gabP* did not improve GABA production. To determine the GABA concentration produced, we characterized the GABA sensor in each EcN strain in the identical growth conditions using exogenously added GABA (Figure S1). When we accounted for the slightly different response of the sensor in each condition and strain, a comparable titer of GABA was produced with or without the deletion of *gabT* and *gabP* and reached 0.053 ± 0.007 mM and 0.06 ± 0.01 mM, respectively, in M9 medium 10 h after induction (Figure S2). Given these results, we chose not to use the EcN Δ*gabTP* strain for subsequent experiments and used the wildtype EcN host instead.

We next examined how the culturing vessel used for growth affects GABA production. Previously, the GABA biosensor was assayed in small-volume cultures using 96-well plates, which are commonly used for sensor characterization [25]. However, this presented challenges in this work, as there was an insufficient cell population to analyze multiple time points, and cultures had low cell densities. To test how scaling up affected GABA biosynthesis, we performed the GABA production assay for the EcN pML3021 production strain in 96-well plates, 14-mL culture tubes, and 125-mL Erlenmeyer flasks. In media at pH 7.0, we again observed insignificant GABA sensor activation and negligible GABA production for all growth vessels (Figure 2E). At pH 5.5, we observed GABA sensor activation beginning 8 h after GadB induction for cultures in plates, tubes, and flasks, and increasing activation through 10 h (Figure 2F). Due to the very fast growth rate of EcN, we could not continue culturing beyond 10 h and maintain exponential growth. When we used the GABA sensor response curves (Figure S1) to determine the GABA concentration produced, interestingly, the amount of GABA was comparable and statistically indistinguishable for all three vessels (Figure S3). However, the flask cultures had the highest reproducibility and lowest background GABA production without GadB induction. Therefore, we chose to use flask cultures in subsequent experiments.

### 3.2. Feedback Control of GABA Production Rate

**Figure 3:**
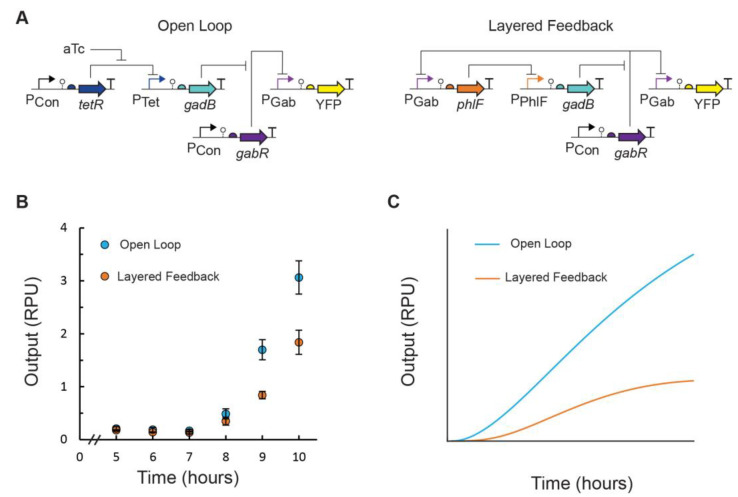
EcN GABA production controlled by open-loop and layered-feedback genetic circuits. (**A**) In the open-loop control scheme (pML3021), GadB expression is induced by an exogenous inducer and produces GABA, which activates the P_Gab_ sensor output promoter of the GABA biosensor. The P_Gab_ signal is measured using an insulated eYFP transcriptional unit. In the layered-feedback control scheme (pML3032), the P_Gab_ output of the GABA biosensor is the input to a transcriptional NOT gate (P_PhlF_ repressed by PhlF), which regulates GadB expression and GABA production detected by the GABA biosensor. (**B**) GABA production assays were performed for EcN cells containing plasmids pML3021 (blue) and pML3032 (orange). Cells were inoculated (OD_600_ = 5 × 10^−5^) in M9 media with MSG supplementation at pH 5.5 in 125 mL Erlenmeyer flasks. For cells containing the open-loop circuit, 2 ng/mL aTc inducer was added. After 5 h of growth, cell fluorescence was measured via flow cytometry every hour. Fluorescence was converted into relative promoter units (RPU). Markers represent the average of the measured median of a population of at least 10,000 cells assayed in three identical experiments performed on three separate days. Error bars represent the standard deviation. (**C**) Simulation predictions of dynamic GABA production for the open-loop (blue) and layered-feedback (orange) designs from the corresponding mathematical model for each (Methods). Output is reported as the P_Gab_ sensor output in RPU.

After constructing our EcN GABA production strain, we next sought to further engineer this strain to integrate dynamic regulation of GABA biosynthesis via synthetic regulatory networks. To compare circuit topologies, we constructed genetic circuits with open-loop control (identical to our inducible GABA biosynthesis system) and layered-feedback control, in which a negative feedback loop was created using a characterized repressor-based NOT gate, which acts as a transcriptional signal inverter (Figure 3A). In the latter design, the inducible promoter input expressing *gadB* (P_Tet_) was replaced by the repressible promoter P_Phlf_, which is the output promoter of the PhlF NOT gate [22]. The expression of the PhlF repressor was placed under the control of P_Gab_. In this way, the GABA sensor output feeds into the genetic circuit, which in turn regulates the expression of GadB and GABA biosynthesis, creating the closed-loop feedback. The GABA production of EcN cells transformed with the constructed plasmids was assayed in flasks using the conditions determined above and the GABA biosensor readout. We observed a slower rate of GABA production for cells containing the layered-feedback circuit compared to the open loop (Figure 3B). This is consistent with predictions obtained from our mathematical models (Figure 3C) and in agreement with similar feedback loops reported in the literature [17,18]. We observed detectable GABA production for both designs beginning 8 h after inoculation. While we also predicted a lower concentration of GABA at steady state for the layered-feedback control, this could not be confirmed experimentally here using this batch growth system, given that growth could not be extended beyond 10 h.

**Figure 4:**
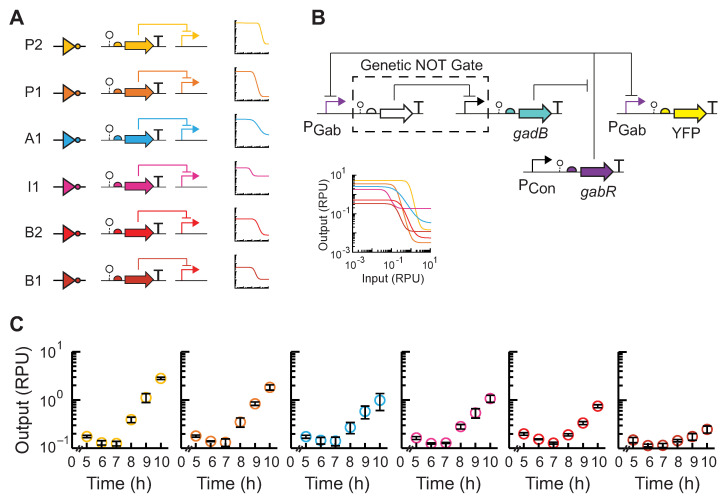
Tunable layered feedback using modular repressor NOT gates to control GABA production. (**A**) Six repressor-based NOT gates (Table S3), each containing a TetR-family repressor and its cognate promoter, were selected. The circuit NOT gate symbol, genetic schematic, and empirical response function in EcN is shown for each. Colors indicate the NOT gate identity. The overlay of the gate response functions is shown for comparison. (**B**) Each NOT gate was integrated into the closed-loop layered-feedback circuit, and the corresponding plasmid designs were constructed. (**C**) GABA production assays were performed for EcN cells transformed with each feedback circuit plasmid. Cultures were inoculated (OD_600_ = 5 × 10^−5^) and grown in M9 supplemented with MSG at pH 5.5 in 125 mL Erlenmeyer flasks. After 5 h of growth, cell fluorescence was measured via flow cytometry every hour. Fluorescence was converted into relative promoter units (RPU) of the P_Gab_ sensor promoter. Markers represent the average of the measured median of a population of at least 10,000 cells assayed in three identical experiments performed on three separate days. Error bars represent the standard deviation. Colors indicate the NOT gate identity shown in (**A**).

Lastly, we posited that the substitution of the repressor NOT gate in the layered-feedback circuit with another could modulate the feedback and be used as a control strategy to achieve a desired rate of production and steady state output of GABA, as supported by modeling predictions (Figure 1C). For this experiment, we utilized a library of insulated repressor NOT gates [21] (Figure 4A). Across this set of six NOT gates, four repressors (PhlF, AmtR, IcaRA, and BM3R1) were chosen, and two gates contained an RBS variant for the repressor (PhlF, BM3R1) (Table S3). We next constructed the five additional layered-feedback circuits, each containing a different NOT gate that has a unique response function in EcN (Figure 4B). EcN cells were transformed with each plasmid construct and assayed for GABA production (Figure 4C). As expected, we observed large differences in the rate of GABA production among the set of feedback circuits and up to an 11-fold difference in output at 10 h. We observed rough agreement with our predicted outputs from mathematical models containing the transfer function of each gate with the qualitative trends and rank order (Figure 1C and Figure 4C).

## 4. Discussion

Engineered probiotic bacteria provide an opportunity to locally sense and control biomolecules in the gastrointestinal tract that would otherwise prove difficult to manipulate. Here, we utilized an intracellular sensor for the neurotransmitter GABA [25] to engineer and evaluate GABA production in EcN under various conditions, accelerating a rate-limiting analytical step via high-performance liquid chromatography [26]. In this work, we found that the deletion of *gabTP* did not improve GABA biosynthesis and that physiologically relevant concentrations of GABA could be produced by overexpressing only the GadB glutamate decarboxylase. While we found GadB expression alone to be sufficient for GABA production, this approach and the GABA biosensor could be applied for further metabolic engineering to enhance GABA production, such as facilitating the screening of large libraries of GadB mutants or combinatorial pathway engineering of GadA, GadB, and GadC [27]. This work also provides a demonstration of using biosensor-guided metabolic engineering, here shown in the clinically relevant EcN bacterium.

Using feedback in gene-regulatory networks, other researchers have been able to predictably and robustly control a range of cellular outputs [17,18]. Here, we build upon these works and implement closed-loop control via layered negative feedback to self-regulate GABA production in engineered EcN. We show that by utilizing an intracellular sensor and modular genetic circuit components, we can design and create feedback circuits to control GABA production and its dynamics. By utilizing a library of repressor logic gates in the feedback circuit, we show, computationally and experimentally, that we can alter and tune the dynamics of GABA biosynthesis. Further study is warranted to determine the long-term dynamics of GABA biosynthesis in the engineered EcN strains and to determine whether cellular transport may limit the extracellular delivery of GABA. Looking further ahead, many further experiments are anticipated in order to understand the potential clinical viability of this approach, such as understanding the effects of expected in vivo perturbations on feedback control, the effects of variable pH along the GI tract on GABA biosynthesis, and how microbial cells can effectively deliver synthesized GABA in vivo. However, this work provides a step toward designing synthetic self-regulation of microbial metabolism for GABA biosynthesis in a gut microbe. The biosensor-assisted screening strategy presented here could accelerate the investigation of these perturbations on GABA production. Moreover, the approach of using modular transcriptional logic gates to construct and tune the dynamics of feedback circuits may be broadly applied for in situ control of metabolite biosynthesis across microbiota and microbiomes.

## Data Availability

The raw data supporting the conclusion of this article will be made available by the authors without undue reservation. Data is not publicly available due to privacy.

## Supplementary Materials

The following supporting information can be downloaded at: https://www.mdpi.com/article/10.3390/metabo14010044/s1, Figure S1: GABA sensor characterization in production conditions. The GABA sensor characterization plasmid pML3009 was induced with exogenous GABA at each concentration and grown in the following strains and vessels. (A) EcN Δ*gabTP* pML3009 grown in flasks, (B) EcN pML3009 grown in flasks, (C) EcN pML3009 grown in culture tubes, and (D) EcN pML3009 grown in a 96-well microtiter plate. All cultures were inoculated at OD600 = 5.0 × 10^−5^. Single cell fluorescence was measured via flow cytometry (Methods). The markers represent the average of the median fluorescence measured on 3 separate days. The error bars are one standard deviation. The fitted response function for each is shown (solid line); Figure S2: GABA production in EcN and EcN Δ*gabTP*. For the data of the GABA production assays in Figure 2D, the output of the GABA biosensor was converted to GABA concentration using the corresponding fitted sensor response function (Figure S1). The bars represent the mean value from the 3 experiments and the error bars represent the standard deviation; Figure S3: EcN GABA production in different growth vessels. For the data of the GABA production assays in Figure 2F, the output of the GABA biosensor was converted to GABA concentration using the corresponding fitted sensor response function (Figure S1). The bars represent the mean value from the 3 experiments and the error bars represent the standard deviation. Values of 0 mM are displayed as 0.000011 mM on the plot; Table S1: Genetic part sequences used in this work; Table S2: Plasmids used in this work; Table S3: Hill equation parameters for NOT gates; Table S4: Model parameter values used [21,22,23,25,33,36,37,38].
