# Supplementary material for: Genetic Circuits for Feedback Control of Gamma-Aminobutyric Acid Biosynthesis in Probiotic Escherichia coli Nissle 1917
Source: Metabolites. 2024 Jan 11;14(1):44. doi: 10.3390/metabo14010044 (PMC10819706; doi:10.3390/metabo14010044)
Supplement: Supplementary file 1 [file metabolites-14-00044-s001.zip › metabolites-2778298-supplementary.pdf]

Supplementary Materials for

Genetic circuits for feedback control of gamma-aminobutyric acid biosynthesis in probiotic *Escherichia coli* Nissle 1917

Matthew Lebovich[1,2], Marcos A. Lora[1], Jared Gracia-David[1,3], and Lauren B. Andrews[1,2,3*]

[1] University of Massachusetts Amherst, Department of Chemical Engineering, Amherst, MA 01003, USA
[2] University of Massachusetts Amherst, Biotechnology Training Program, Amherst, MA, USA
[3] Amherst College, Department of Biology, Amherst, MA 01002, USA
[4] University of Massachusetts Amherst, Molecular and Cellular Biology Graduate Program, Amherst, MA, USA

* Correspondence: lbandrews@umass.edu

Contents

Figure S1: GABA sensor characterization in production conditions.. 3
Figure S2: GABA production in EcN and EcN *ΔgabTP*... 4
Figure S3: EcN GABA production in different growth vessels 5
Table S1: Genetic part sequences used in this work.. 6
Table S2: Plasmids used in this work 12
Table S3: Hill equation parameters for NOT gates 13
Table S4: Model parameter values used 14
References... 15

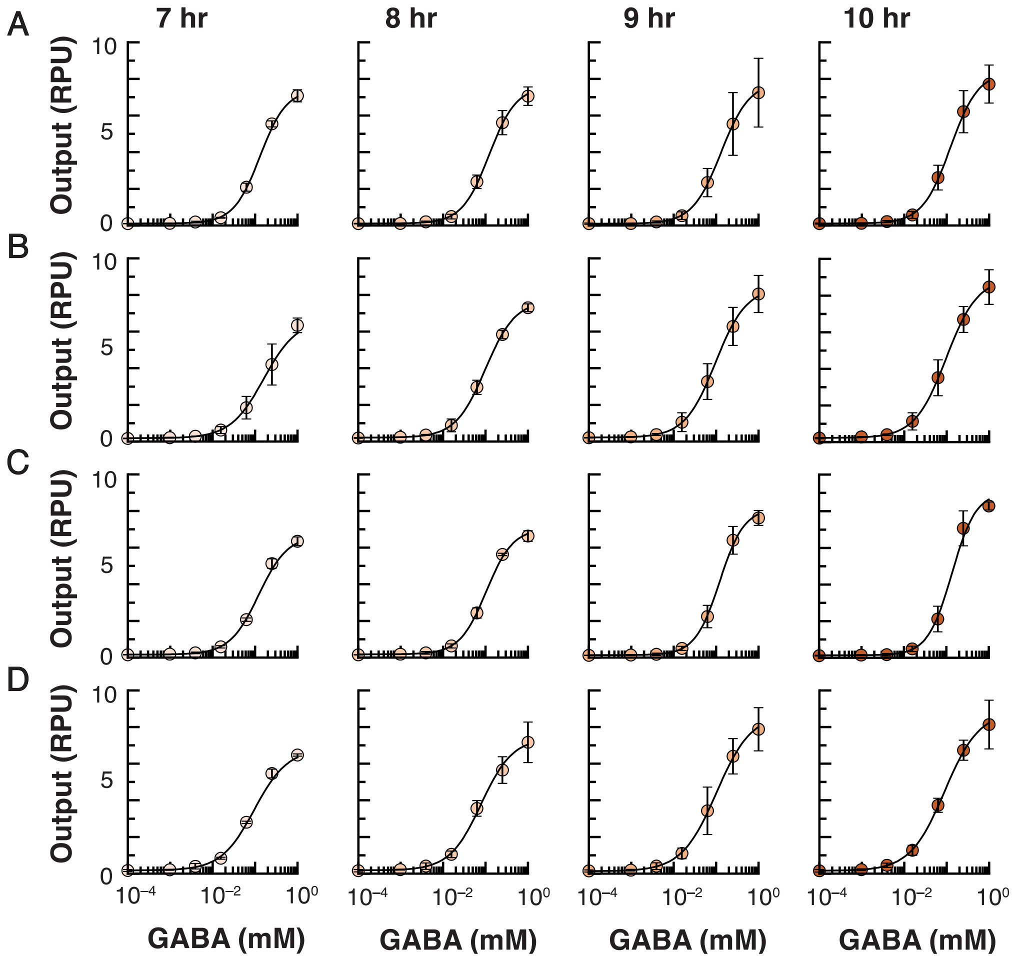

Figure S1: GABA sensor characterization in production conditions. The GABA sensor characterization plasmid pML3009 was induced with exogenous GABA at each concentration and grown in the following strains and vessels. **(A)** EcN Δ*gabTP* pML3009 grown in flasks, **(B)** EcN pML3009 grown in flasks**, (C)** EcN pML3009 grown in culture tubes, and **(D)** EcN pML3009 grown in a 96-well microtiter plate. All cultures were inoculated at OD_{600} = 5.0 × 10^{-5}. Single cell fluorescence was measured via flow cytometry (Methods). The markers represent the average of the median fluorescence measured on 3 separate days. The error bars are one standard deviation. The fitted response function for each is shown (solid line).

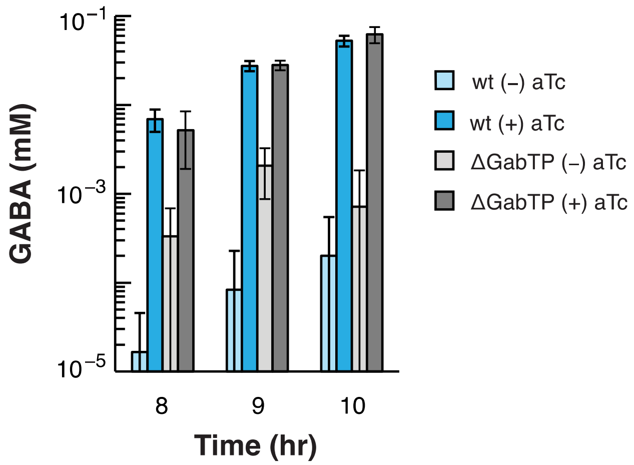

Figure S2: GABA production in EcN and EcN *ΔgabTP*. For the data of the GABA production assays in Figure 2D, the output of the GABA biosensor was converted to GABA concentration using the corresponding fitted sensor response function (Figure S1). The bars represent the mean value from the 3 experiments and the error bars represent the standard deviation.

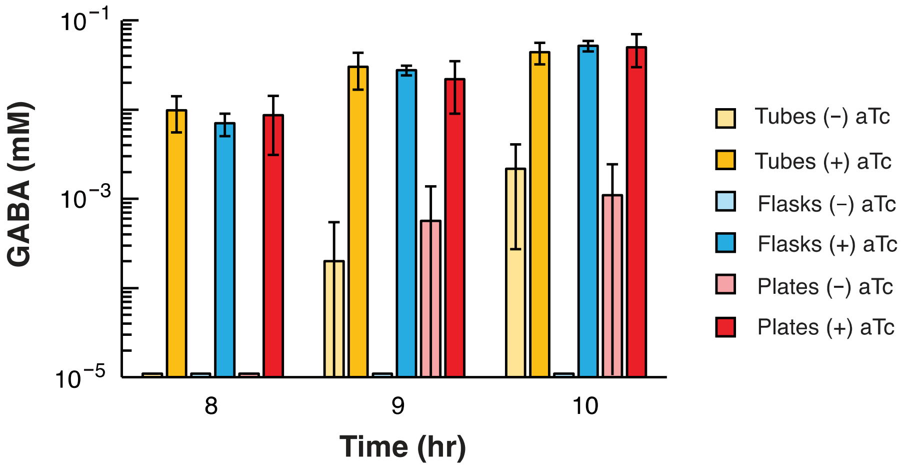

Figure S3: EcN GABA production in different growth vessels. For the data of the GABA production assays in Figure 2F, the output of the GABA biosensor was converted to GABA concentration using the corresponding fitted sensor response function (Figure S1). The bars represent the mean value from the 3 experiments and the error bars represent the standard deviation. Values of 0 mM are displayed as 0.000011 mM on the plot.

Table S1: Genetic part sequences used in this work

Part Name	Type	DNA Sequence	Source
P_{AmtR}	Promoter	cttgtccaaccaaatgattcgttaccaattgacagtttctatcgatctata gataatgctagc	(22)
P_{BM3RI}	Promoter	Aatccgcgtgataggtctgattcgttaccaattgacggaatgaacgttc attccgataatgctagc	(22)
P_{IcaRA}	Promoter	gtcaactcataagattctgattcgttaccaattgacaattcacctacctttc gttaggttaggttgt	(22)
P_{PhlF}	Promoter	cgacgtacggtggaatctgattcgttaccaattgacatgatacgaaac gtaccgtatcgttaaggt	(22)
P_{Gab105}	Promoter	ataccatcaaaaagttataattggtactttacggcataccaagtcctagg tactatgctagc	(25)
P_{Tet}	Promoter	Tactccaccgttggctttttttccctatcagtgatagagattgacatccct atcagtgatagagataatgagcac	
A1	RBS	aatgttccctaataatcagcaaagaggttactag	(21)
B1	RBS	ctatggactatgttttaactactag	(21)
B2	RBS	ctatggactatgtttttcaaagacgaaaaactactag	(21)
I1	RBS	attgctatggactatgtttcaaagtgagaatactag	(21)
P1	RBS	ctatggactatgtttgaaagggagaaatactag	(21)
P2	RBS	ggagctatggactatgtttgaaaggctgaaatactag	(21)
amtR	Gene	atggcaggcgcagttggtcgtccgcgtcgtagtgcaccgcgtcgtgc aggtaaaaatccgcgtgaagaaattctggatgcaagcgcagaactgtt tacccgtcagggttttgcaaccaccagtacccatcagattgcagatgc agttggtattcgtcaggcaagcctgtattatcattttccgagcaaaaccg aaatctttctgaccctgctgaaaagcaccgttgaaccgagcaccgttct ggcagaagatctgagcaccctggatgcaggtccggaaatgcgtctgt gggcaattgttgcaagcgaagttcgtctgctgctgagcaccaaatgga atgttggtcgtctgtatcagctgccgattgttggtagcgaagaatttgca gaatatcatagccagcgtgaagcactgaccaatgtttttcgtgatctgg caaccgaaattgttggtgatgatccgcgtgcagaactgccgtttcatatt	(22)

		accatgagcgttattgaaatgcgtcgcaatgatggtaaaattccgagtc<br>cgctgagcgcagatagcctgccggaaaccgcaattatgctggcagat<br>gcaagcctggcagttctgggtgcaccgctgcctgcagatcgtgttgaa<br>aaaaccctggaactgattaaacaggcagatgcaaaataa	
bm3RI	Gene	atggaaagcaccccgaccaaacagaaagcaattttagcgcaagcct<br>gctgctgtttgcagaacgtggttttgatgcaaccaccatgccgatgatt<br>gcagaaaatgcaaaagttggtgcaggcaccatttatcgctatttcaaaa<br>acaaagaaagcctggtgaacgaactgtttcagcagcatgttaatgaatt<br>tctgcagtgtattgaaagcggtctggcaaatgaacgtgatggttatcgt<br>gatggctttcatcacatttttgaaggtatggtgacctttaccaaaaatcat<br>ccgcgtgcactgggttttatcaaaacccatagccagggcacctttctga<br>ccgaagaaagccgtctggcatatcagaaactggttgaatttgtgtgca<br>cctttttcgtgaaggtcagaaacagggtgtgattcgtaatctgccgga<br>aaatgcactgattgcaattctgtttggcagctttatggaagtgtatgaaat<br>gatcgagaacgattatctgagcctgaccgatgaactgctgaccggtgt<br>tgaagaaagcctgtgggcagcactgagccgtcagagctaa	(22)
icaRA	Gene	gtgaaagacaaaattatcgataacgccatcaccctgtttagcgaaaaa<br>ggttatgacggcaccaccctggatgatattgcaaaaagcgtgaacatc<br>aaaaaagccagcctgtattatcactttgatagcaaaaaaagcatctacg<br>agcagagcgttaaatgctgtttcgattatctgaacaacatcatcatgatg<br>aaccagaacaaaagcaactatagcatcgatgccctgtatcagtttctgt<br>ttgagttcatcttcgatatcgaggaacgctatattcgtatgtatgttcagct<br>gagcaacacaccggaagaattttcaggtaacatttatggccagatcca<br>ggatctgaatcagagcctgagcaaagaaatcgccaaattctatgacg<br>aaagcaaaatcaaaatgaccaaagaggacttccagaatctgattctgc<br>tgtttctggaaagctggtatctgaaagccagctttagccagaaatttggt<br>gcagttgaagaaagcaaaagccagtttaaagatgaggtttatagcctg<br>ctgaacatctttctgaagaaataa	(22)
phlF	Gene	atggcacgtaccccgagccgtagcagcattggtagcctgcgtagtcc<br>gcatacccataaagcaattctgaccagcaccattgaaatcctgaaaga<br>atgtggttatagcggtctgagcattgaaagcgttgcacgtcgtgccggt<br>gcaagcaaaccgaccatttatcgttggtggaccaataaagcagcact<br>gattgccgaagtgtatgaaaatgaaagcgaacaggtgcgtaaatttcc<br>ggatctgggtagctttaaagccgatctggattttctgctgcgtaatctgt<br>ggaaagtttggcgtgaaaccatttgtggtgaagcatttcgttgtgttattg<br>cagaagcacagctggaccctgcaaccctgacccagctgaaagatca<br>gtttatggaacgtcgtcgtgagatgccgaaaaaactggttgaaaatgc<br>cattagcaatggtgaactgccgaaagataccaatcgtgaactgctgct<br>ggatatgatttttggtttttgttggtatcgcctgctgaccgaacagctgac<br>cgttgaacaggatattgaagaatttaccttcctgctgattaatggtgtttg<br>tccgggtacacagcgttaa	(22)

gabR	Gene	atggatatcacgattacactcgatcgttcagaacaagccgattatatcta<br>tcagcaaatttatcaaaagctgaaaaaagaaatcctcagccgcaatct<br>gctgccgcactcgaaggttccctccaagcgggagctggctgaaaatc<br>tcaaggtcagcgtaaattcagtgaattcagcctatcagcagctgctgg<br>ctgaggggtatttgtacgccattgaacgaaagggtttcttcgtggagga<br>actagacatgttttccgccgaggagcaccctccatttgcactgccggat<br>gacctaaaagagattcacatcgaccagagcgattggatatcgttttcac<br>acatgagttccgatacagaccattttccgatcaaaagctggttccgctg<br>cgagcaaaaagcggcctcccgctcataccgcacgctcggcgatatgt<br>cacatccgcaagggatatatgaagtgagagcggccattacgaggctc<br>atttccctgacgaggggtgtaaaatgcaggccggaacaaatgatcata<br>ggggcaggcacacaggtgctcatgcagctgttgactgagcttttaccc<br>aaggaagccgtgtatgcgatggaggagcctggctacaggcgcatgt<br>atcagcttttgaagaatgccggaaaacaagtaaagacgatcatgctgg<br>atgaaaaaggcatgtcgattgctgaaatcaccagacagcagccagat<br>gtgctggtgaccaccccgtcgcatcagtttccgtccggaacgattatg<br>cctgtatccagaagaattcagctgctgaactgggcagccgaggagcc<br>gcgccgatatatcattgaggacgattatgatagtgaattcacatatgatg<br>tagacagtattccggcgctgcaaagcctcgaccgttttcaaaatgtcat<br>ctatatgggaaccttttcaaagtcccttctccccggcttacggatcagct<br>atatggtgttgccgcctgagctgttgagggcatacaaacagcggggc<br>tatgatctgcagacttgctcatcactcacacagctcaccctgcaggaat<br>ttatcgagtctggtgaatatcagaagcatataaaaaaaatgaagcagc<br>attataaagaaaagagagaacgcctgatcaccgctttagaagcagag<br>ttcagcggagaggttaccgtaaaaggggcaaatgcggggctgcattt<br>tgttaccgaatttgataccaggcgcaccgaacaagacatcctgtcaca<br>tgctgccgggctgcagcttgaaatattcggaatgagccgatttaacttg<br>aaggaaaacaagcggcaaacgggcaggcctgctctcattatcggctt<br>tgcacggctgaaggaagaagatattcaggagggtgtgcagcggcttt<br>tcaaagcggtttacggacataaaaaaatccccgttacaggggattga	(36)
eYFP	Gene	atggtgagcaagggcgaggagctgttcaccggggtggtgcccatcc<br>tggtcgagctggacggcgacgtaaacggccacaagttcagcgtgtc<br>cggcgagggcgagggcgatgccacctacggcaagctgaccctgaa<br>gttcatctgcaccacaggcaagctgcccgtgccctggcccaccctcg<br>tgaccaccttcggctacggcctgcaatgcttcgcccgctaccccgacc<br>acatgaagctgcacgacttcttcaagtccgccatgcccgaaggctac<br>gtccaggagcgcaccatcttcttcaaggacgacggcaactacaagac<br>ccgcgccgaggtgaagttcgagggcgacaccctggtgaaccgcatc<br>gagctgaagggcatcgacttcaaggaggacggcaacatcctggggc<br>acaagctggagtacaactacaacagccacaacgtctatatcatggcc<br>gacaagcagaagaacggcatcaaggtgaacttcaagatccgccaca<br>acatcgaggacggcagcgtgcagctcgccgaccactaccagcaga	(37)

		acaccccaatcggcgacggccccgtgctgctgcccgacaaccacta ccttagctaccagtccgccctgagcaaagaccccaacgagaagcgc gatcacatggtcctgctggagttcgtgaccgccgccgggatcactctc ggcatggacgagctgtacaagtaa	
lacI	Gene	atgaaaccagtaacgttatacgatgtcgcagagtatgccggtgtctctt atcagaccgtttcccgcgtggtgaaccaggccagccacgtttctgcga aaacgcgggaaaaagtggaagcggcgatggcggagctgaattacat tcccaaccgcgtggcacaacaactggcgggcaaacagtcgttgctg attggcgttgccacctccagtctggccctgcacgcgccgtcgcaaatt gtcgcggcgattaaatctcgcgccgatcaactgggtgccagcgtggt ggtgtcgatggtagaacgaagcggcgtcgaagcctgtaaagcggcg gtgcacaatcttctcgcgcaacgcgtcagtgggctgatcattaactatc cgctggatgaccaggatgccattgctgtggaagctgcctgcactaatg ttccggcgttatttcttgatgtctctgaccagacacccatcaacagtatta ttttctcccatgaggacggtacgcgactgggcgtggagcatctggtcg cattgggtcaccagcaaatcgcgctgttagcgggcccattaagttctgt ctcggcgcgtctgcgtctggctggctggcataaatatctcactcgcaat caaattcagccgatagcggaacgggaaggcgactggagtgccatgt ccggttttcaacaaaccatgcaaatgctgaatgagggcatcgttccca ctgcgatgctggttgccaacgatcagatggcgctgggcgcaatgcgc gccattaccgagtccgggctgcgcgttggtgcggatatctcggtagtg ggatacgacgataccgaagatagctcatgttatatcccgccgttaacc accatcaaacaggattttcgcctgctggggcaaaccagcgtggaccg cttgctgcaactctctcagggccaggcggtgaagggcaatcagctgtt gccagtctcactggtgaaaagaaaaaccaccctggcgcccaatacg caaaccgcctctccccgcgcgttggccgattcattaatgcagctggca cgacaggtttcccgactggaaagcgggcagtga	(22)
tetR	Gene	atgtccagattagataaaagtaaagtgattaacagcgcattagagctgc ttaatgaggtcggaatcgaaggtttaacaacccgtaaactcgcccaga agctaggtgtagagcagcctacattgtattggcatgtaaaaaataagc gggctttgctcgacgccttagccattgagatgttagataggcaccatac tcacttttgccctttagaaggggaaagctggcaagattttttacgtaata acgctaaaagttttagatgtgctttactaagtcatcgcgatggagcaaa agtacatttaggtacacggcctacagaaaaacagtatgaaactctcga aaatcaattagcctttttatgccaacaaggtttttcactagagaatgcatt atatgcactcagcgctgtggggcattttactttaggttgcgtattggaag atcaagagcatcaagtcgctaaagaagaaagggaaacacctactact gatagtatgccgccattattacgacaagctatcgaattatttgatcacca aggtgcagagccagccttcttattcggccttgaattgatcatatgcgga ttagaaaaacaacttaaatgtgaaagtgggtcctaa	(22)

kanR	Gene	atgagccatattcaacgggaaacgtcttgctccaggccgcgattaaattccaacatggatgctgatttatatgggtataaatgggctcgcgataatgtcgggcaatcaggtgcgacaatctatcgattgtatgggaagcccgatgcgccagagttgtttctgaaacatggcaaaggtagcgttgccaatgatgttacagatgagatggtcagactaaactggctgacggaatttatgcctcttccgaccatcaagcattttatccgtactcctgatgatgcatggttactcaccactgcgatccccgggaaaacagcattccaggtattagaagaatatcctgattcaggtgaaaatattgttgatgcgctggcagtgttcctgcgccggttgcattcgattcctgtttgtaattgtccttttaacagcgatcgcgtatttcgtctcgctcaggcgcaatcacgaatgaataacggtttggttgatgcgagtgattttgatgacgagcgtaatggctggcctgttgaacaagtctggaaagaaatgcataagcttttgccattctcaccggattcagtcgtcactcatggtgatttctcacttgataaccttatttttgacgaggggaaattaataggttgtattgatgttggacgagtcggaatcgcagaccgataccaggatcttgccatcctatggaactgcctcggtgagttttctccttcattacagaaacggctttttcaaaaatatggtattgataatcctgatatgaataaattgcagtttcatttgatgctcgatgagtttttctaa	(33)
BydvJ	Insulator	gggtgtctcaaggtgcgtaccttgactgatgagtccgaaaggacgaaacacccctctacaaataattttgtttaa	(21)
ElvJ	Insulator	gccccatagggtggtgtgtaccacccctgatgagtccaaaaggacgaaatggggcctctacaaataattttgtttaa	(21)
SarJ	Insulator	gactgtcgccggatgtgtatccgacctgacgatggcccaaaagggccgaaacagtcctctacaaataattttgtttaa	(21)
RiboJ53	Insulator	gcggtcaacgcatgtgctttgcgttctgatgagacagtgatgtcgaaaccgcctctacaaataattttgtttaa	(21)
RiboJ64	Insulator	aggagtcaattaatgtgcttttaattctgatgagacggtgacgtcgaaactccctctacaaataattttgtttaa	(21)
L3S2P55	Terminator	ctcggtaccaaagacgaacaataagacgctgaaaagcgtcttttttcgtttttggtcc	(38)
L3S2P24	Terminator	ctcggtaccaaattccagaaaagacacccgaaagggtgttttttcgttttggtcc	(38)
ECK120015170	Terminator	acaattttcgaaaaaacccgcttcggcgggtttttttatagctaaaa	(38)
L3S2P11	Terminator	ctcggtaccaaattccagaaaagagacgctttcgagcgtcttttttcgttttggtcc	(38)
ECK120033737	Terminator	ggaaacacagaaaaaagcccgcacctgacagtgcgggcttttttttcgaccaaagg	(38)

L3S2P11	Terminator	ctcggtaccaaattccagaaaagagacgctttcgagcgtcttttttcgttttggtcc	(38)
ECK120033737	Terminator	ggaaacacagaaaaaagcccgcacctgacagtgcgggctttttttttcgaccaaagg	(38)
L3S3P21	Terminator	ccaattattgaaggcctccctaacggggggcctttttttgtttctggtctccc	(38)
L3S3P51	Terminator	aaaaaaaaaaaacaccctaacgggtgttttttgtttctggtctccc	(38)

Table S2: Plasmids used in this work

Plasmid Name	Description	Source
pAN1717	RPU strain expressing YFP under control of J23101 on a backbone expressing *lacI*, *tetR*, *kanR* and the p15a ori	(21)
pML3001	Plasmid backbone containing g*abR*, *lacI* and *tetR* as well as the resistance gene *kanR* and the p15a ori.	(25)
pML3009	Plasmid expressing YFP under the control of P_{Gab} on the pML3001 backbone	(25)
pML3021	Open loop GABA production circuit. P_{Gab}-YFP, P_{Tet}-GadB	This work
pML3030	Feedback circuit using the IcaRA I1 NOT gate on the pML3001 backbone	This work
pML3031	Feedback circuit using the AmtR A1 NOT gate on the pML3001 backbone	This work
pML3032	Feedback circuit using the PhlF P1 NOT gate on the pML3001 backbone	This work
pML3033	Feedback circuit using the PhlF P2 NOT gate on the pML3001 backbone	This work
pML3034	Feedback circuit using the BM3RI B1 NOT gate on the pML3001 backbone	This work
pML3035	Feedback circuit using the BM3RI B2 NOT gate on the pML3001 backbone	This work

Table S3: Hill equation parameters for NOT gates

**Repressor**	**RBS**	y_{min}	y_{max}	K	n
AmtR	A1	0.032	2.597	0.209	1.881
BM3R1	B1	0.012	0.336	0.178	3.437
BM3R1	B2	0.005	0.517	0.317	2.865
IcaRA	I1	0.187	1.847	0.066	3.772
PhlF	P1	0.003	3.550	0.175	3.924
PhlF	P2	0.015	5.306	0.843	4.880

All parameters are from a prior study (23).

Table S4: Model parameter values used

Parameter	Value	Units
α_B	1.0	[GadB]/([RNA]*min)
γ	0.025	1/min
β_B	0.10	1/min
β_{μ_G}	20.0	1/min
ξ	0.025	[mRNA]/(min*RPU)
k	0.25	1/min
S	35.0	g/L
β_G	3.0	g/(L*min)
α_{μ_G}	3.0	RPU/min
$y_{min\mu_G}$	0.04	RPU
$y_{max\mu_G}$	4.59	RPU
K_{μ_G}	16.23	g/L
$n_{\mu G}$	0.90	unitless

Parameters γ and ξ are based on a prior study (33).

Parameters $y_{min\mu_G}$, $y_{max\mu_G}$, K_{μ_G}, and $n_{\mu G}$ are based on a prior study (25).

References Cited in Supplementary Materials

(21) Nielsen, A. A. K.; Der, B. S.; Shin, J.; Vaidyanathan, P.; Paralanov, V.; Strychalski, E. A.; Ross, D.; Densmore, D.; Voigt, C. A. Genetic Circuit Design Automation. *Science* 2016, 352, aac7341. https://doi.org/10.1126/science.aac7341.

(22) Stanton, B. C.; Nielsen, A. A. K.; Tamsir, A.; Clancy, K.; Peterson, T.; Voigt, C. A. Genomic Mining of Prokaryotic Repressors for Orthogonal Logic Gates. *Nat. Chem. Biol.* 2014, 10 (2), 99–105. https://doi.org/10.1038/nchembio.1411.

(23) Lebovich, M.; Zeng, M.; Andrews, L. B. Algorithmic Programming of Sequential Logic and Genetic Circuits for Recording Biochemical Concentration in a Probiotic Bacterium. *ACS Synth. Biol.* 2023, 12 (9), 2632–2649. https://doi.org/10.1021/acssynbio.3c00232.

(25) Lebovich, M.; Andrews, L. Surveying the Genetic Design Space for Transcription Factor-Based Metabolite Biosensors: Synthetic Gamma-Aminobutyric Acid and Propionate Biosensors in *E. coli* Nissle 1917. *Front. Bioeng. Biotechnol.* 2022, 10, 1227. https://doi.org/10.3389/fbioe.2022.938056.

(33) Andrews, L. B.; Nielsen, A. A. K.; Voigt, C. A. Cellular Checkpoint Control Using Programmable Sequential Logic. *Science* 2018, 361, eaap8987. https://doi.org/10.1126/science.aap8987.

(36) Belitsky, B. R.; Sonenshein, A. L. GabR, a Member of a Novel Protein Family, Regulates the Utilization of γ-Aminobutyrate in *Bacillus subtilis*. *Mol. Microbiol.* 2002, 45 (2), 569–583. https://doi.org/10.1046/j.1365-2958.2002.03036.x.

(37) Cormack, B. P.; Valdivia, R. H.; Falkow, S. FACS-Optimized Mutants of the Green Fluorescent Protein (GFP). *Gene* 1996, 173 (1), 33–38. https://doi.org/10.1016/0378-1119(95)00685-0.

(38) Chen, Y.-J.; Liu, P.; Nielsen, A. A. K.; Brophy, J. A. N.; Clancy, K.; Peterson, T.; Voigt, C. A. Characterization of 582 Natural and Synthetic Terminators and Quantification of Their Design Constraints. *Nat. Methods* 2013, 10 (7), 659–664. https://doi.org/10.1038/nmeth.2515.
